# Ciprofloxacin-Resistant *Neisseria meningitidis*, Delhi, India

**DOI:** 10.3201/eid1310.060820

**Published:** 2007-10

**Authors:** Smita Singhal, Kedar P. Purnapatre, Vandana Kalia, Smita Dube, Deepti Nair, Monorama Deb, Pushpa Aggarwal, Sunil Gupta, Dilip J. Upadhyay, Ashok Rattan, V. Samuel Raj

**Affiliations:** * Ranbaxy Research Laboratories, Gurgaon, India; † Vardhman Mahaveer Medical College and Safdarjung Hospital, New Delhi, India; ‡ Ministry of Health and Family Welfare, New Delhi, India; § National Institute of Communicable Diseases, Delhi, India; ¶ Caribbean Epidemiology Centre, Port of Spain, Trinidad and Tobago

**Keywords:** Ciproflaxin, antimicrobial resistance, meningitis, India, dispatch

## Abstract

Decreased susceptibility of *Neisseria meningitidis* isolates to ciprofloxacin emerged from an outbreak in Delhi, India. Results of antimicrobial susceptibility testing of the meningococcal isolates to ciprofloxacin and further sequencing of DNA gyrase A quinolone-resistance–determining region confirmed the emergence of ciprofloxacin resistance in the outbreak.

*Neisseria meningitidis* serogroup A is the major cause of meningitis outbreaks worldwide, especially in African and Asian countries, including India. Meningococcal disease is endemic in India, and sporadic cases of meningococcal meningitis have occurred in Delhi in previous years (*1*). During 1966, 616 cases of meningitis were reported; case-fatality rate was 20.9%. In 1985, an outbreak of greater magnitude had 6,133 cases with 799 deaths (13%). An outbreak of meningococcal meningitis also occurred in Delhi during April–July 2005 (*1*), and the disease reappeared in January–March 2006.

When a sporadic case or epidemic occurs, the close contacts need to receive a vaccine and chemoprophylaxis with antimicrobial drugs to cover the delay between vaccination and protection. Recently, ciprofloxacin and ceftriaxone have been established as acceptable alternatives to rifampin for prophylaxis of meningococcal disease (*2*). Because ciprofloxacin can be used in single doses during meningococcal epidemics, ciprofloxacin is the chemoprophylactic agent of choice (*3*). Because meningococcal disease is a serious and rapidly progressing illness, monitoring the trends in the resistance to antimicrobial agents is important. To date, ciprofloxacin-resistant serogroup A *N. meningitidis* has not been reported anywhere in the world. Four reports of sporadic instances of decreased susceptibility to ciprofloxacin: serogroup B from France in 1999 and Spain in 2002, serogroup C from Australia in 1998, and serogroup Y from Argentina in 2002 (*4**–**6*). For the first time, to our knowledge, we report the emergence of decreased susceptibility of serogroup A *N. meningitidis* to ciprofloxacin from the 2005 outbreak in Delhi.

## The Study

A total of 444 meningococcal cases and 62 deaths due to meningococcal meningitis serogroup A were reported in Delhi during April–July 2005. The reappearance of meningococcal cases was reported in Delhi during January–March 2006 (177 meningococcal cases and 17 deaths). The meningococcal cases were reported from the major hospitals and were characterized by sudden onset of fever with petechial rash, neck stiffness, and altered sensory functions. All age groups were affected, but the highest proportion was in those 15–30 years of age; male patients accounted for 71% of cases and female patients 29%.

Fourteen *N. meningitidis* clinical isolates were collected from the major hospitals in Delhi. All these isolates were from patients with meningococcal meningitis from the outbreak. The strains were isolated mainly from cerebrospinal fluid and a few from skin swabs; the primary culture, isolation, and serogrouping (Latex agglutination kit, Wellcogen, Wellcome, Dartford, UK) were performed in the respective hospitals. *N. meningitidis* strains from Ranbaxy Research Laboratories culture collection (RRL-1 and RRL-2) were used as reference serogroup A strains. In addition, 11 clinical isolates collected during the January–March 2006 recurrent outbreak were also included for selective antimicrobial drug susceptibility testing in this study.

The MICs of antimicrobial agents against these isolates were determined by the agar dilution method on Mueller-Hinton agar plates with 5% sheep blood as recommended by Clinical and Laboratory Standard Institute (CLSI) guidelines (*7*). The pulsed-field gel electrophoresis (PFGE) method used in this study was based on the procedures described by Popovic et al. (*8*). Two isolates (Ap-II 420 and Ir-I 442) from the outbreak were used for genotyping as described by Maiden et al. (*9*) and analyzed by using the multilocus sequence typing (MLST) database (*10*).

Because the *N. meningitidis* isolates showed resistance to ciprofloxacin, the DNA gyrase A quinolone-resistance–determining regions (QRDRs) of 2 ciprofloxacin-sensitive and 7 ciprofloxacin-resistant meningococcal strains from this outbreak and 2 reference strains were sequenced. The following primers were used for QRDR study. The forward primer was 5′-CGTACTGTACGCGATGCACGA-3′; the reverse primer was 5′-TTTCGCCATGCGGATTTCGGT-3′. The genomic DNA was isolated from the strains and PCR was performed as described by Shultz et al. (*11*).

The MIC pattern of the clinical isolates of *N. meningitidis* from the outbreak showed that they were susceptible to β-lactam antibiotics penicillin, ampicillin, and a third-generation cephalosporin, ceftriaxone. The MIC against ceftriaxone for all the clinical *N. meningitidis* isolates was <0.001 µg/mL. Of 14 strains, 12 showed decreased susceptibility to all the fluoroquinolones, especially to ciprofloxacin (MIC 0.25 µg/mL) (Appendix Table). In addition, all these 12 strains were resistant to nalidixic acid (MIC >16 µg/ml). A notable shift in antimicrobial susceptibility was not observed in these isolates against protein synthesis inhibitors (Appendix Table). Eleven meningococcal isolates from the recurrent outbreak showed resistance to ciprofloxacin as well as nalidixic acid (Table). The break points are based on the CLSI guidelines (*7*).

**Table:** Antimicrobial susceptibility pattern of *Neisseria meningitidis* isolates from the recurrent outbreak during January– March 2006, Delhi, India*

Isolate	MIC (μg/mL)									
	PEN	CRO	RIF	CIP	GAT	MXF	LVX	SPX	NOR	NAL
SFDJ M-1	0.125	0.008	0.004	0.125	0.03	0.125	0.125	0.06	0.125	>16
SFDJ M-2	0.125	0.008	0.004	0.25	0. 25	0.125	0.03	0.06	0.25	>16
SFDJ 691	0.03	<0.001	0.125	0.25	0.25	0.5	0.25	0.25	0.5	>16
SFDJ 306	0.03	0.008	0.008	0.125	0.06	0.125	0.25	0.06	0.25	>16
SFDJ 651	0.06	0.002	0.03	0. 5	0.125	0.25	0.25	0.125	0.5	>16
SFDJ 568	0.125	0.002	0.06	0.25	0.125	0.25	0.25	0.125	0.5	>16
SFDJ 668	0.06	0.002	0.06	0.25	0.125	0.25	0.125	0.125	0.25	>16
SFDJ 270	0.125	0.008	0.015	0.125	0.06	0.125	0.125	0.125	0.25	>16
SFDJ MA-3	0.06	0.004	0.125	0.5	0.25	0.25	1	1	1	>16
SFDJ 339	0.125	<0.001	0.25	0.25	0.25	0.25	0.25	0.25	0.25	>16
SFDJ 58	0.125	0.008	0.004	0.125	0.06	0.125	0.03	0.06	0.25	>16

*PEN, penicillin; CRO, ceftriaxone; RIF, rifampin; CIP, ciprofloxacin; GAT, gatifloxacin; MFX, moxifloxacin; LVX, levofloxacin; SPX, sparfloxacin; NOR, norfloxacin; NAL, nalidixic acid.

**Figure 1:**
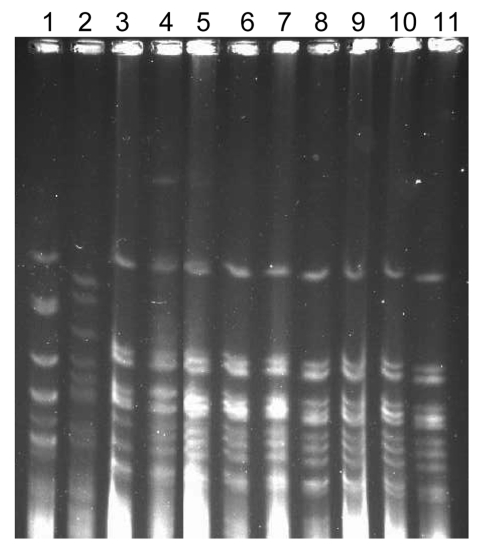
Pulsed-field gel electrophoresis (PFGE) pattern of the *Neisseria meningitidis* isolates from the outbreak. The chromosomal DNA digested with SpeI enzyme was separated by clamped homogeneous electric fields PFGE (BioRad, Hercules, CA, USA). 1, RRL-1; 2, RRL-2; 3, SFDJ 723; 4, Ap-II 420; 5, SFDJ E-95; 6, IR-I 442, 7, IR-II 440; 8, SFDJ E-100; 9, SFDJ 184; 10, SFDJ E-79; 11, NICD 18.

The PFGE patterns of the strains from the outbreak (9 strains) were indistinguishable, and they appeared to be from the same origin (Figure 1). The antimicrobial drug resistance patterns were not distinguishable from the PFGE types. The PFGE pattern of the strains from the outbreak was different from that of the reference strains RRL-1 and RRL-2. The housekeeping genes of the ciprofloxacin-resistant strain (Ir-I 442) showed no alteration or mutation, and they were identical to the ciprofloxacin-sensitive strain (Ap-II 420). *Neisseria* MLST analysis with the MLST database showed that these isolates were similar to the outbreak strains from Dhaka, Bangladesh (2002), and Nigeria (2003) (*10*). In addition to the endemicity in Delhi, the migration of persons or visitors from other countries may have contributed to the spread of the serogroup A outbreak.

**Figure 2:**
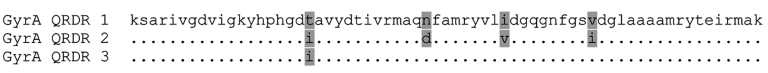
Comparison of GyrA quinolone-resistance–determining region (QRDR) for the alteration of amino acids in *Neisseria meningitidis* isolates. GyrA QRDR 1, ciprofloxacin-sensitive strains RRL-1, RRL-2, SFDJ 723, Ap-II 420; GyrA QRDR 2, ciprofloxacin-resistant strains SFDJ E-100, SFDJ E-79, SFDJ E-63, NICD 18, SFDJ E-95, IR-I 442; GyrA QRDR 3, ciprofloxacin-resistant strain IR-II 440. Ciprofloxacin-sensitive RRL-1 and RRL-2 were the reference strains, and the other 9 strains were from the outbreak. The shaded positions of the amino acids denote changes in GyrA QRDR of ciprofloxacin-resistant *N. meningitidis* in Thr-91 → Ile, Asn-103 → Asp, Ile-111 → Val, and Val-120 → Ile. The mutation of amino acid at conservative position 91 (Thr-91 → Ile) is responsible for the decreased susceptibility to fluoroquinolones, and the other 3 changes are synonymous.

The sequencing of the *gyrA* QRDRs of ciprofloxacin-resistant strains of *N. meningitidis* showed 4 amino acid differences. One of these encoded a conservative threonine substitution at position 91 (Thr-91 → Ile); the other 3 changes were synonymous: Asn-103 → Asp, Ile-111 → Val, and Val-120 → Ile (Figure 2). Gyrase A changes have been reported in clinical isolates of resistant meningococci in positions 91 (Thr-91 → Ile) and 95 (Asp-95 → Asn and Asp-95 → Gly) (*4*–6). The *N. meningitidis gyrA* gene shares 95% identity with the *N. gonorrhoeae gyrA* gene. The GyrA substitution in meningococci at position 91 (Thr-91 → Ile) was equivalent to Ser-91 → Ile, reported in fluoroquinolone-resistant *N. gonorrhoeae* from Japan (*12*). In meningococci as well as in gonococci, the mutation at position 91 causes fluoroquinolone resistance. For example, 1 *N. meningitidis* strain from 2005 outbreak, IR-II 440, showed the mutation only at position 91 (Thr-91 → Ile), and ciprofloxacin resistance was observed.

## Conclusions

The antimicrobial drug susceptibility of the clinical isolates and further sequencing of the QRDRs of gyrase A of the strains confirms the emergence of ciprofloxacin resistance in the outbreak in Delhi. In addition to chemoprophylaxis, fluoroquinolone consumption in the community, for a range of infections may be, in part, responsible for the emergence of ciprofloxacin resistance in *N. meningitidis* isolates. Lack of *N. gonorrhoeae* isolate response to ciprofloxacin has been reported in Delhi (*13*). Drift in susceptibility of *N. gonorrhoeae* to ciprofloxacin caused therapeutic failure. Ciprofloxacin treatment failure in cases of typhoid fever has also been reported (*14*). To date, to our knowledge, no failure of ciprofloxacin as chemoprophylaxis for *N. meningitidis* has been reported. To predict clinical outcome, further detailed pharmacokinetic/pharmacodynamic (PK/PD) analysis is needed to determine the impact of the PK/PD parameters on resistance selectivity (*15*).

## Supplementary Material

Appendix Table Antimicrobial susceptibility pattern of Neisseria meningitidis isolates from the outbreak during April-July 2005 Delhi, India*
