# Supplementary material for: Ciprofloxacin-Resistant Neisseria meningitidis, Delhi, India
Source: Emerg Infect Dis. 2007 Oct;13(10):1614–6. doi: 10.3201/eid1310.060820 (PMC2851515; doi:10.3201/eid1310.060820)
Supplement: Appendix Table — Antimicrobial susceptibility pattern of Neisseria meningitidis isolates from the outbreak during April-July 2005 Delhi, India* [file 06-0820_appT-s1.pdf]

Appendix Table. Antimicrobial susceptibility pattern of *Neisseria meningitidis* isolates from the outbreak during April–July 2005 Delhi, India*

Isolate	MIC (μg/mL)																
	PEN	AMP	CRO	RIF	CIP	GAT	MXF	OFL	NOR	NAL	ERY	CLR	AZM	TEL	TET	MIN	COT
SFDJ 723	0.06	0.06	<0.001	0.125	0.008	0.004	0.03	0.015	0.015	0.25	2	0.5	0.5	0.25	2	0.25	16
SFDJ E-100	0.06	0.06	<0.001	0.125	0.25	0.125	0.25	0.5	0.5	>16	2	0.5	0.5	0.25	2	0.25	>16
AP-II 420	0.06	0.06	<0.001	0.125	0.015	0.008	0.03	0.015	0.015	0.25	1	0.5	0.5	0.25	2	0.5	>16
IR-II 440	0.06	0.06	<0.001	0.06	0.25	0.125	0.25	0.5	0.5	>16	2	0.5	0.5	0.25	2	0.25	>16
SFDJ 184	0.06	0.06	<0.001	0.125	0.25	0.125	0.25	0.5	0.5	>16	2	0.5	0.5	0.5	4	0.25	>16
SFDJ 137	0.06	0.06	<0.001	0.125	0.25	0.125	0.25	0.5	0.5	>16	2	0.5	1	0.25	4	0.25	>16
SFDJ E-79	0.06	0.06	<0.001	0.125	0.25	0.125	0.25	0.5	0.5	>16	2	0.5	0.5	0.25	4	0.25	>16
SFDJ E-63	0.06	0.06	<0.001	0.125	0.25	0.125	0.25	0.5	0.5	>16	2	0.5	0.5	0.25	4	0.25	>16
NICD 18	0.03	0.06	<0.001	0.125	0.25	0.125	0.25	0.5	0.5	>16	2	0.5	0.5	0.25	4	0.25	>16
SFDJ E-95	0.06	0.06	<0.001	0.125	0.25	0.125	0.25	0.5	0.5	>16	2	0.5	0.5	0.25	4	0.25	>16
SFDJ E-57	0.06	0.06	<0.001	0.125	0.25	0.125	0.25	0.5	0.5	>16	2	0.5	0.5	0.25	4	0.25	>16
SFDJ E-11	0.06	0.06	<0.001	0.125	0.25	0.125	0.125	0.5	0.5	>16	2	0.5	0.5	0.25	4	0.25	>16
IR-I 442	0.06	0.06	<0.001	0.125	0.25	0.125	0.125	0.5	0.5	>16	2	0.5	0.5	0.25	4	0.25	>16
NICD *22*	0.06	0.06	<0.001	0.125	0.25	0.125	0.25	0.5	0.5	>16	2	0.5	0.5	0.25	2	0.25	>16
RRL-1	0.06	0.06	<0.001	0.008	0.008	0.004	0.03	0.015	0.03	0.25	0.5	0.06	0.125	0.06	0.125	0.06	1
RRL-2	0.06	0.06	<0.001	0.004	0.008	0.008	0.06	0.015	0.008	0.25	0.25	0.06	0.125	0.06	0.125	0.06	1

*PEN, penicillin; AMP, ampicillin; CRO, ceftriaxone; RIF, rifampin; CIP, ciprofloxacin; GAT, gatifloxacin; MXF, moxifloxacin; OFL, ofloxacin; NOR, norfloxacin; NAL, nalidixic acid; ERY, erythromycin; CLR, clarithromycin; AZM, azithromycin; TEL, telithromycin; TET, tetracycline; MIN, minocycline; COT, cotrimoxazole.
